# A robust estimation of exon expression to identify alternative spliced genes applied to human tissues and cancer samples

**DOI:** 10.1186/1471-2164-15-879

**Published:** 2014-10-08

**Authors:** Alberto Risueño, Beatriz Roson-Burgo, Anna Dolnik, Jesus M Hernandez-Rivas, Lars Bullinger, Javier De Las Rivas

**Affiliations:** Bioinformatics and Functional Genomics Research Group, Cancer Research Center (CiC-IBMCC, CSIC/USAL/IBSAL), Salamanca, 37007 Spain; Instituto de Investigación Biomédica de Salamanca (IBSAL), Salamanca, 37007 Spain; Department of Internal Medicine III, University Hospital of Ulm, Ulm, 89081 Germany; Servicio de Hematología y Departamento de Medicina, Hospital Universitario de Salamanca (HUS), Salamanca, 37007 Spain; Celgene Institute for Translational Research Europe (CITRE), Sevilla, Spain

**Keywords:** Alternative splicing, Splicing index, Human genomics, Exons, Transcripts, Gene expression, Differential expression, Bioinformatics, R algorithm, Acute myeloid leukemia

## Abstract

**Background:**

Accurate analysis of whole-gene expression and individual-exon expression is essential to characterize different transcript isoforms and identify alternative splicing events in human genes. One of the *omic* technologies widely used in many studies on human samples are the exon-specific expression microarray platforms.

**Results:**

Since there are not many validated comparative analyses to identify specific splicing events using data derived from these types of platforms, we have developed an algorithm (called ESLiM) to detect significant changes in exon use, and applied it to a reference dataset of 270 human genes that show alternative expression in different tissues. We compared the results with three other methodological approaches and provided the R source code to be applied elsewhere. The genes positively detected by these analyses also provide a verified subset of human genes that present tissue-regulated isoforms. Furthermore, we performed a validation analysis on human patient samples comparing two different subtypes of acute myeloid leukemia (AML) and we experimentally validated the splicing in several selected genes that showed exons with highly significant signal change.

**Conclusions:**

The comparative analyses with other methods using a fair set of human genes that show alternative splicing and the validation on clinical samples demonstrate that the proposed novel algorithm is a reliable tool for detecting differential splicing in exon-level expression data.

**Electronic supplementary material:**

The online version of this article (doi:10.1186/1471-2164-15-879) contains supplementary material, which is available to authorized users.

## Background

The human transcriptome presents a high degree of complexity that most recent research on cell transcriptomics is trying to unravel [1]. Individual human genes often produce multiple mRNAs and protein isoforms through alternative processing of their pre-mRNA. This process of RNA “alternative splicing” increases the size and diversity of the proteome by generating different gene products per locus. The isoforms produced by alternative splicing may have related or quite distinct functions [2]. In last decade the splicing process has begun to be better understood, revealing a highly controlled regulation [3] and the involvement of a complex molecular machinery [4]. Moreover, this process has not only been associated with normal biological states, but also with pathological states and diseases such as cancer [5, 6]. Despite the progress on our knowledge of splicing events, there is not yet a detailed identification and mapping of all the alternative expression products derived from each human gene locus in different cell types. Recent advances in genomic and transcriptomic technologies have generated large datasets corresponding to expression studies on human genes in different contexts, including signal information at exon level. Analysis of this type of data with accurate measurement of whole-gene expression and individual-exon expression is essential in order to identify alternative splicing events. One of the most frequently applied technologies aimed at this type of studies are the exon-specific expression microarray platforms [7], widely used for example in the ENCODE project (http://genome.ucsc.edu/encode/).

Here, we present a new computational data analysis strategy to achieve a robust calculation of gene and exon expression using such exon-specific platforms and we applied it to detect splicing in a collection of human tissues. The approach is validated with a previously published reference set of 270 human genes that undergo splicing [8], and we compared the results with three other methods also designed to analyze genome-wide exon expression data. The indicated reference set of genes used for validation stems from a study of human tissue transcriptomes obtained by deep-sequencing (NGS) of complementary DNA fragments, which provides an inventory of human genes and mRNA isoforms expression [8]. Our approach validated many of the human genes found to undergo alternative splicing in different tissues in this reference set. Finally, in addition to the study on human tissues, we also applied our novel splicing analysis approach to an independent dataset of human samples from patients with primary acute myeloid leukemia (AML). This analysis allowed the identification of alternatively spliced isoforms that were related with high confidence to two distinct AML subgroups, and for a selected set of genes findings were confirmed experimentally by conventional RT-PCR.

## Methods

### Human tissues expression datasets and human splicing reference genes

**Figure 1:**
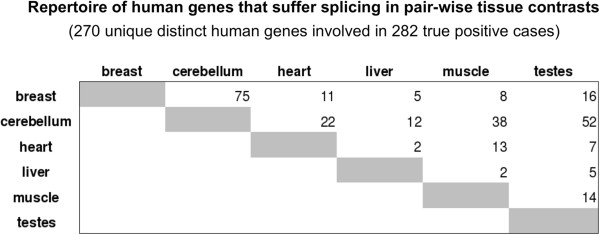
**Repertoire of human genes that undergo splicing in different tissues.** The figure presents the numbers corresponding to 15 pair-wise comparisons between 6 human tissues: breast, cerebellum, heart, liver, muscle, testes. A total of 270 distinct human genes present alternative splicing between these tissues, giving a compendium of 282 true positive cases that are used to evaluate the performance of alternative splicing detection methods.

A dataset of 33 exon microarrays (model GeneChip Human Exon 1.0 ST, *Affymetrix, Inc.*) containing 3 replicas of 11 different healthy human tissues was obtained from http://www.affymetrix.com. This dataset was used to compare the performance in the detection of alternative splicing of the new algorithm proposed here with three other algorithms previously published, using in all cases a reference set of 270 human genes known to undergo splicing (obtained from [8]). The comparison of the tissues present in the exon array dataset and in the splicing dataset of Wang *et al.*[8], returned 6 tissues (breast, cerebellum, heart, liver, muscle and testes) which were analyzed together since they present both exon expression data (from the arrays) and splicing data (from the sequencing). The combination of these 6 tissues, in pairwise comparisons, provides 15 different tissue-pairs. The number of genes reported by Wang *et al*. [8] that are alternatively spliced and were found in those pairwise tissue contrasts was 282 (that correspond to 270 distinct human genes) as indicated in Figure 1. The complete list of these genes is provided in Additional file 1: Table S1.

### Method design and implementation

The method presented in this work, called ESLiM (*Exon Splicing by Linear Modeling Analysis*), has been implemented in R (http://cran.r-project.org/). The method uses the algorithm RMA (from R library *affy*) [9, 10] for the calculation of the expression signal at probe level for each exon and gene loci; and the algorithm LIMMA (from R library *limma*) for the differential expression analyses [11]. The complete method, as an R package, together with the mapping CDF files are provided as supporting data files: (Supporting data 1) ESLiMc R package: *ESLiM_1.0.tar.gz*; (Supporting data 2) Example of Use for ESLiMc R package: *ESLIM_Install_and_Use.R*; (Supporting data 3) ExonMapper CDF R package: *exonmapperhumanexon1.0cdf_3.0.tar*; (Supporting data 4) ESLiMc GeneMapper CDF: *eslimcgenemapperhumanexon1.0cdf_3.0.tar.gz*. The exon annotation package (*exons.human.Annotation.RData*) and the complete raw dataset of 33 exon microarrays (*affy_dataset.zip*), that are quite large files (>20 MB), are available (together with the other supporting data files) at http://bioinfow.dep.usal.es/xgate/splicing/splicing.php.

The methodological motivation was to design an analytical strategy for alternative splicing detection able to calculate the relationship between expression of each specific exon and the corresponding whole gene expression in a robust way. This approach overcomes several problems frequently described in genome-wide expression analyses: **(i)** the difficulty to achieve an accurate calculation of the whole signal corresponding to the gene versus the partial signals corresponding to transcripts or exons in the same locus, since estimation of these partial signals is very much affected when the genes show very different levels of expression along the compared samples; **(ii)** the distortion in the signal values due to artificial “probe effects” in the arrays [9, 10]; and **(iii)** the “cross-hybridization effects” provoked by non-specific probes that detect several gene loci giving ambiguous signals [12].

**Figure 2:**
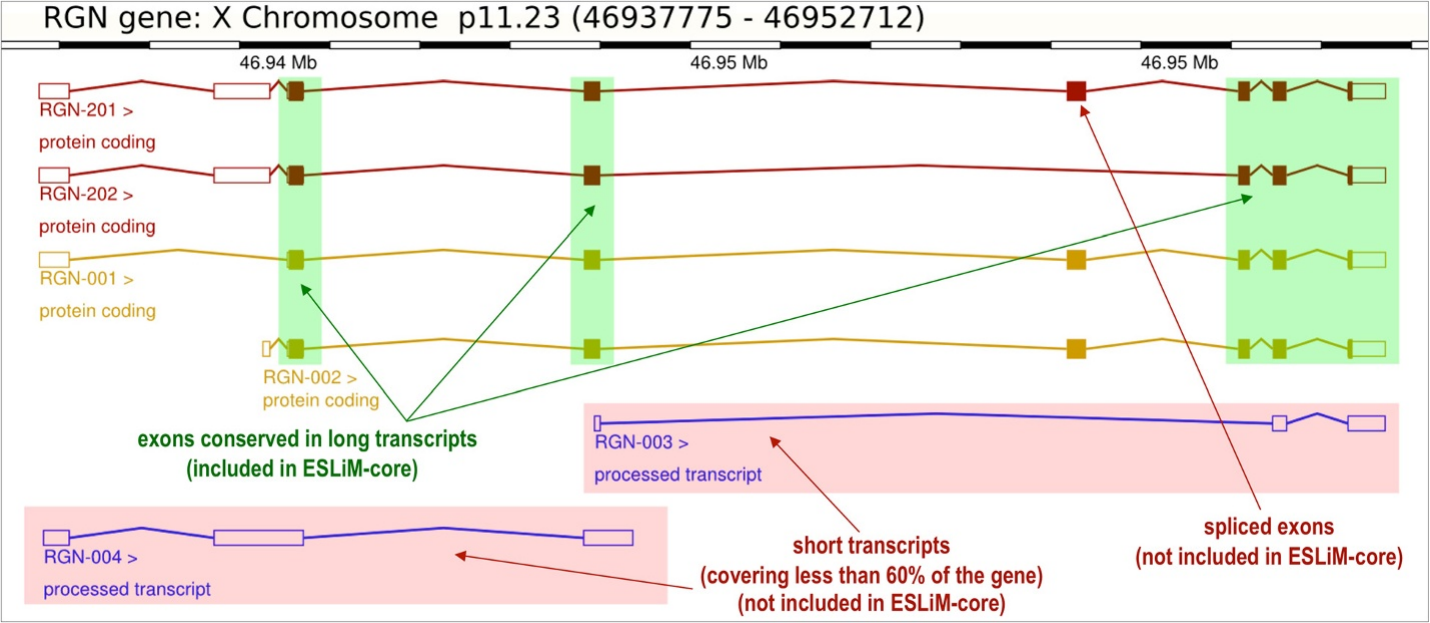
**Scheme of** ***RGN*** **gene locus.** The human gene locus *RGN* (regucalcin, Ensembl ID: ENSG00000130988, located on chromosome X, band p11.23) includes 6 different transcripts (as defined in *Homo sapiens* genome, Ensembl v74): RGN-001, RGN-002, RGN-003, RGN-004, RGN-201 and RGN-202. The transcripts cover different parts of the locus and include different exons (represented as rectangular boxes). Colored boxes correspond to protein-coding sequence. The whole RGN gene locus includes 15 different exons. The green shadows include the exons that are kept common in the long transcripts. The pink shadows include two short transcripts that cover less than 60% of the gene locus. At the top of the figure a black and white bar indicates the position of each exon.

These problems were addressed in the method ESLiM, proposed here, following some strategic criteria: **(a)** provide and use a stringent definition of “gene core” to calculate the gene signal (as described in Figure 2); **(b)** use an approach for the expression calculations based on linear regression models that combines analysis of the signal from multiple samples for each gene-exon pair (this criteria allows to obtain best-performing metrics when using multiple arrays assessed simultaneously) [13]; **(c)** use an unambiguous mapping of all probes to the currently known expressed biological entities, i.e. to the known human protein-coding gene loci and to each specific exon defined on the loci (taking the mapping provided by GATExplorer) [14]. Exon junction probes are not considered in this study for two reasons: **(i)** the generation of exon arrays that we are analyzing are no designed to have exon-exon junction probes because they are based on genome sequence and on the location of exons and introns as defined in the reference human genome (i.e. the probes on the array were not designed to map on cDNAs); **(ii)** the identification of alternative splicing events is based on the individual precise measurement of each exon with specific probes plus the correct estimation of the whole gene expression. The exon-junction probes would be ambiguous for one specific exon and, as indicated above, in the analyses only “non-ambiguous probes” have been considered.

A key rationale behind the method proposed is that, in the absence of alternative splicing events, an increase in the global expression of a gene should correspond to a higher expression in all its exons. Having datasets with multiple samples we can test such correspondence since the data allow the establishment of a relationship between the signals of each gene and the signals of each exon. Such a relationship can be modeled using linear regression analysis. In the formula below, the expected expression  of the exon *i* that belongs to gene *j* and biological sample *k*, is linearly dependent on the expression of the gene *g* following a slope *s*. Each exon-gene pair will have its own slope depending on the accumulated characteristics of their detecting probes:


In this context, a significant difference between the *observed* value and the *expected* value can be interpreted as being due to an alternative splicing event. These differences are called residuals, and graphically represent the deviation of each sample with respect to the linear regression:


The model can be completed with these residuals resulting in the following formula:


Given that this model is performed in a totally unsupervised way, a further supervised test should be done to find the statistically significant differences between residuals belonging to two compared biological or clinical categories (i.e. between two classes). This step of the method was implemented using *limma*[11].

The algorithm developed (ESLiM) included three slightly different versions with respect to how the signals corresponding to the genes were calculated. The first one, named ESLiM-all (**ESLiMa**), uses all the probes located along the gene locus. The second one, named ESLiM-total (**ESLiMt**), considers only the probes intersecting all the known transcripts of the gene locus. Finally, ESLiM-core (**ESLiMc**) is an intermediary approach that, for the calculation of each gene signal, does not consider the small transcripts covering less than 60% of the gene locus (as described in Figure 2).

### Comparative analyses of different methods to detect alternative splicing

The performance of the three approaches, ESLiMa, ESLiMc and ESLiMt, was compared with three other methods: FIRMA [15], ARH [16] and COSIE [17]. All the methods were run in R and applied to the same sample datasets. As indicated above, we used the set of 270 human genes known to undergo splicing as a reference [8]. The differences between scores per exon obtained for each of the 15 tissue pairs compared were assessed with *limma* making a ranking of significance based on the p-values. In the case of ARH the scores for each exon were obtained using the entropy criterion provided by this method. Finally, ROC curves were calculated using the R package *ROCR*[18].

### Independent clinical dataset used for validation

Diagnostic bone marrow or peripheral blood samples were collected from 64 adult patients with either complex karyotype (CK-AML, n = 40) or core binding factor (CBF-AML, n = 24) AML, and peripheral blood samples were obtained from six healthy volunteers. Written informed consent was acquired from all patients and volunteers. Total RNA was extracted from samples that had been enriched for leukemic blasts/mononuclear cells (>85% in all analyzed cases) using Trizol reagent (*Invitrogen*, Carlsbad, CA). Gene expression profiling was performed on *Affymetrix* GeneChip Exon 1.0 ST microarrays (n = 64) inserting 0.2 μg total RNA according to the manufacturer’s protocols (*Affymetrix*, Santa Clara, CA). The complete dataset is available at Gene Expression Omnibus (accession number GSE21337, at http://www.ncbi.nlm.nih.gov/geo/).

### ESLiMc identified splicing events validated by RT-PCR

Twelve selected top candidate genes identified by ESLiMc to undergo alternative splicing were further evaluated by conventional RT-PCR. In brief, 1.0 μg RNA of the diagnostic AML samples was transcribed into cDNA using the SuperScript III Kit with random hexamer primers according to the manufacturer’s recommendations (*Invitrogen*, Carlsbad, CA). Then, cDNA was analyzed by a standard PCR protocol (PCR conditions: 40 cycles with 40s at 94°C, 40s at 55°C, and 60s at 72°C). As a proof of concept and singular testing of the method, two of the most significant genes –within the top 12 selected– were taken for validation of the specific exons that undergo splicing. The following primers that were able to amplify both spliced and unspliced transcript variants (*MAPK15* sense 5′-GCTGCCTTCTAGGACACCTG-3′, *MAPK15* as 5′-CTGGTTGGCCACCTGAGC-3′, *PLXNB1* sense 5′-GGGGGTGTAACTGGTGTGTC-3′ *PLXNB1* as 5′-AGGTCGCCTCTTCCAGCTC-3′). PCR products were cloned in TOPO vector (*Invitrogen*, Carlsbad, CA) and sequenced with TOPO-specific primers M13F and M13R using the ABI Ready Reaction Dye Terminator Cycle Sequencing Kit (Applied Biosystems, Foster City, CA). PCR gel images were quantified using software ImageJ (available at http://rsbweb.nih.gov/ij/). Band densities were quantified in ImageJ software and normalized to the PCR band densities of beta-actin loading control. The correlation plots were plotted in GraphPadPrism 6.0. All the procedures performed in the current study were in accordance with the Declaration of Helsinki and all human samples were collected after signed informed consent was obtained as formally approved on by the corresponding Ethics Committee of the University Hospital of Salamanca (HUS), the Cancer Research Center (CiC-IBMCC) and the University Hospital of Ulm.

## Results and discussion

### Human gene loci architecture: search for a consensus gene signal

Before starting the analysis of the expression signal coming from a given transcriptomic platform, we had to consider the general architecture of a human gene locus. Most human genes include multiple exons along their loci that can be used in the alternative transcripts expressed from each specific locus. In many cases, the exact number of alternative transcripts derived from a given gene is not yet known, and the human genome databases include many putative transcripts with quite variable coverage along the locus. This variability and uncertainty provokes serious problems in the calculation of the gene expression signals when using high throughput omic techniques which map all the annotated exons for each locus –such as RNA-seq or exon microarrays–, because the final gene products (i.e. the proteins) may stem only from the expression of a subset of exons. In order to avoid or minimize this important problem, we propose to use only the common “consensus set of exons” that are present in the major transcripts of a given locus for the calculation of the gene expression signal. The “consensus exons” can refer either to the “complete full exons” that are conserved along all the transcripts or to the specific regions or “exonic-segments” conserved. However, in practical terms for this work, exons from two transcripts that have a partial overlapping will only be considered the same exon when they are mapped by the same oligonucleotide probes in the exon microarrays. Therefore, along the analyses the exon conservation will be always measured by the presence of the corresponding probes that map on the specific exons.

In Figure 2 we show an example illustrating the complexity of a locus for the human gene *RGN* (located on chromosome X). For this gene, six different transcripts have been defined as possible expressed entities. Two of these transcripts (RGN-003 and RGN-004) are quite short and cover less than 60% of the whole locus. The other transcripts (RGN-001, RGN-002, RGN-201 and RGN-202) cover most of the locus, and include the protein-coding sequences corresponding to this gene. In this way, these four transcripts have a stable annotation in Ensembl database (with label *protein-coding* and not just *processed transcript*) according to the human reference genome (*Homo sapiens* GRCh37, Ensembl v74). The whole *RGN* gene locus includes 15 different exons to build all these transcripts. Only 5 exons are conserved in the long transcripts (green boxes in Figure 2) and comprise protein-coding sequences.

Considering this complexity observed in the majority of the human gene loci, we propose three possible ways to account for the transcription signal attributed to a given locus: **(i)** to use all the exons defined in each whole locus to calculate the expression signal of the corresponding gene (this is done in method **ESLiM-all**, **ESLiMa**); **(ii)** to use only the common set of exons conserved in all the transcripts (i.e. the “consensus conserved exons”) (this is done in method **ESLiM-total**, **ESLiMt**); **(iii)** to use only the exons conserved in the long transcripts that cover at least 60% of the locus (this is done in method **ESLiM-core**, **ESLiMc**) (Figure 2). We define and test these three different methodological approaches to find which one yields the most robust way to determine alternative splicing. It is important to emphasize that the classical standard methods usually take “all the exons” (i.e. they follow the first approach named here **ESLiM-all**), and so, this is the *by-default* approach that does not consider the architecture of the gene loci. In this work we test this approach comparing it with the other two, **ESLiM-total** and **ESLiM-core**, which are designed to provide a robust and stable estimation of the expression signal of the genes considering the “conservation” of exons in the known transcripts associated to each gene locus. These second and third approaches do this in two different ways. The second method (**ESLiM-total**) considers the exons that are fully conserved in all the transcripts and this can be very stringent, reducing the coverage of genes. In fact, in the example case of gene *RGN*, there is not any exon that is conserved in all its six transcripts (Figure 2), therefore using **ESLiM-total** the signal for this gene will not be measurable. The third method, **ESLiM-core**, looked for a balance between the all exons approach (which can provide “noisy signal” in many cases) and the complete conserved exons approach (which, as indicated, can provide “lack of signal” in many cases). To do so, **ESLiM-core** takes into consideration the exons conserved only in the transcripts that cover a major part of the locus. We fix a coverage threshold of ≥60% of the locus length after analyzing the architecture of all known human protein-coding genes (Additional file 5: Table S7). In this analysis we observed that most (85%) of the transcripts that cover ≥60% of the human loci length correspond to stable and well-annotated protein-coding transcripts. As indicated in Figure 2, the short transcripts correspond many times to mRNAs that are not translated and are called “processed transcripts” in Ensembl database. We also calculate that 99.7% of the human gene loci included at least one protein-coding transcript that covered ≥60% of its locus length. In this way, using this threshold of ≥60%, we lost signal in less than 0.3% of the genes. By contrast, using for example a threshold of ≥75%, 330 loci would be lost (i.e. applying such threshold 1.6% of the human gene loci will not be detectable by the method) (see Additional file 5: Table S7).

### Combined estimation of gene and exon expression signals

As it has been described, in order to detect alternative splicing events in gene loci, we need an adequate calculation and comparison of the expression signals of each gene and its individual exons. These signal measurements should come from transcriptomic platforms able to detect each gene locus and the individual exons included in it. Every platform has its own technical characteristics that should be considered in order to achieve a quantitative calculation of the expression signals. In case of high-density oligonucleotide microarrays designed to measure exon and gene expression, commonly used platforms (such as *Affymetrix* Exon 1.0 ST Arrays) include oligo probes that map to each known exon. The probes can have variable response characteristics [17] and it is important to use updated and accurate mapping of the probes to genes and to exons [14]. The expression of each gene is calculated using the probes that map to the exons along a given gene locus and the expression of each exon is calculated using the specific probes that map to such exon. If a gene is expressed and there is no alternative splicing event, the exons of such a gene should have a signal similar to the average signal of the whole gene (i.e. a non-spliced exon across analyzed samples is expected to show an expression signal correlated with the signal of the whole gene). In this way, the relationship between the expression of every single exon and the corresponding gene can be modeled using a linear regression.

**Figure 3:**
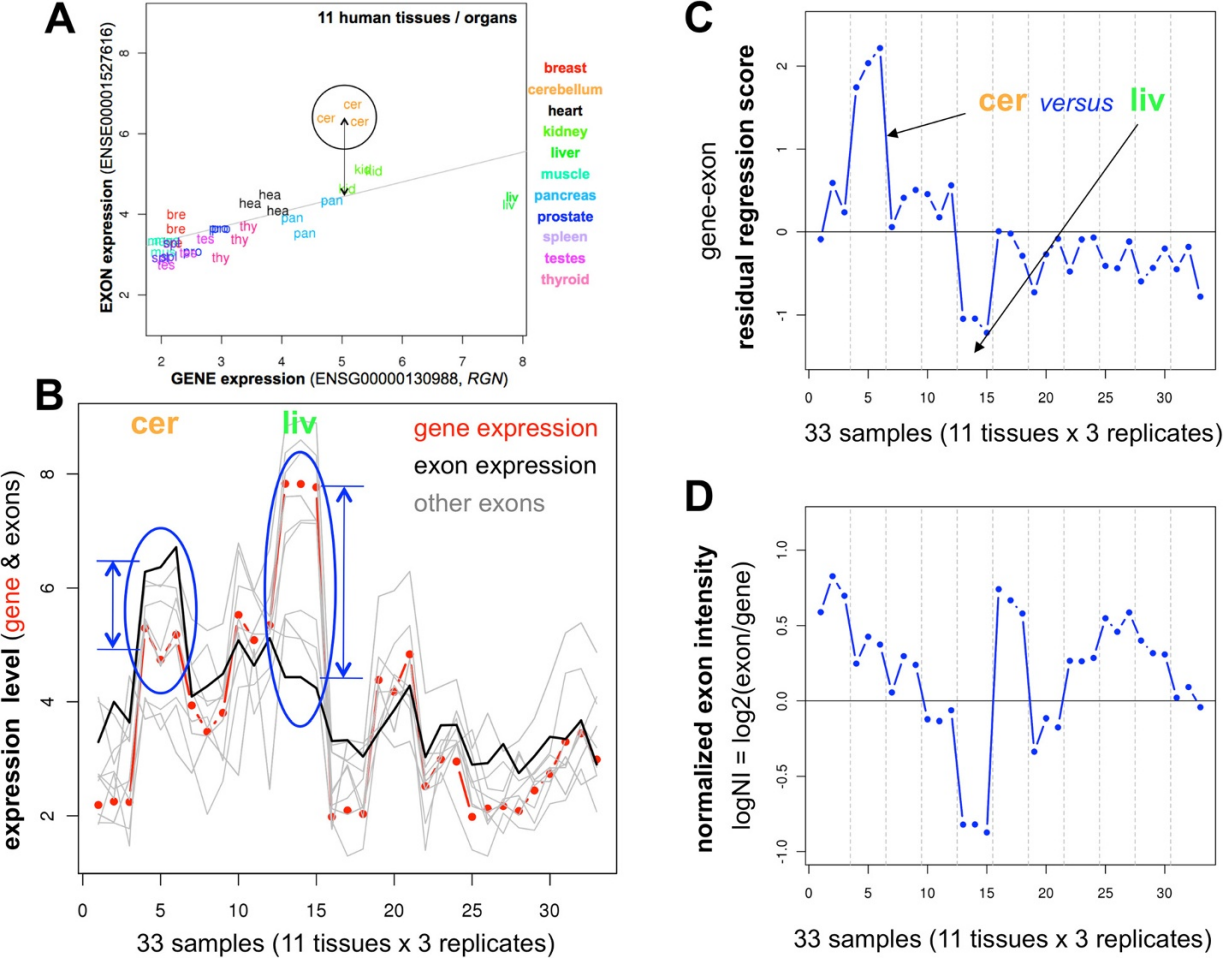
**Exon expression and gene expression.** Calculation of exon expression *versus* gene expression using the standard *splicing index* and the *residual regression score* proposed in this work. **(A)** Plot of the exon expression signal *versus* the gene expression signal of gene *RGN* measured in 11 human tissue samples by exon microarrays (3 replicates per sample). The exon signal is calculated using the probes that map to the specific exon ENSE00001527616 and the gene signal is calculated using the probes that map to the common conserved exons included in the long transcripts of the gene. A linear regression is applied to the data to calculate the expression of the gene-exon pair. The 3 samples corresponding to cerebellum (labeled as “cer”) are placed in a circle to show that the average expression of these samples shows a significant deviation from the linear regression calculated for the whole gene. **(B)** Expression signal profile of gene *RGN* (ENSG00000130988) across the 33 samples (red line with dots) compared to the profile of exon ENSE00001527616 (black line) and the profiles of the rest of the exons (grey lines). **(C)** Plot of the *residual regression scores* calculated for each sample for the gene-exon pair ENSG00000130988-ENSE00001527616. **(D)** Plot of the *splicing index*es calculated for each sample for the gene-exon pair ENSG00000130988-ENSE00001527616.

The exon-gene relationship can be graphically represented considering the expression values, as it is done in Figure 3A. This figure shows the exon expression signal *versus* the gene expression signal of the exemplary gene *RGN* measured in a collection of 11 human tissues samples by exon microarrays (with 3 biological replicates per sample). The exon signal is calculated using only the probes that map to the specific exon (in the example: ENSE00001527616) and the gene signal is calculated using the signal from the probes that map to the common conserved exons included in the long transcripts (as indicated in the previous section). Due to the fact that the human genes measured in different tissues usually show a distribution of expression levels, we can calculate a linear regression between all the measurements to estimate the expected expression signal for this gene-exon pair (Figure 3A). Using this regression analysis, if a given group of samples corresponding to one specific tissue shows a significant deviation of their expression signal with respect to the linear regression of the gene-exon pair, we can estimate that such exon is subjected to alternative splicing in the respective tissue. This is the case for gene *RGN* in the three cerebellum samples (labels “cer” in Figure 3A). If the signal of the tissue samples is over the regression line, the exon will be more expressed (e.g. up-regulated in cerebellum); and if the signal of the tissue samples is below the regression line, the exon will be less expressed (e.g. down-regulated in liver). Following this strategy, we have designed an algorithm that calculates significant differential expression for each exon comparing all the sample classes in a given dataset based on the deviations of the exon signals from the linear regression calculated for the corresponding gene-exon pair. The algorithm is provided as an R tool easy to use. Details about the algorithm and the application guidelines are in the Methods section and in the supporting data section.

### Gene-exon linear regression model compared to splicing index approach

The most common and known approaches to identify alternative splicing are based on the calculation of the *splicing index* (**SI**) [19] or modifications of this parameter. The *splicing index* is defined as the *log*-ratio of the exon intensities between the two sample types after normalization to the gene intensities in each sample: **SI**_**i**_ = log_2_( (e_1i_/g_1j_) / (e_2i_/g_2j_) ) , for the ***i-th*** exon of the ***j-th*** gene in sample type 1 or 2. If a given exon provides the same signal as the whole gene, the exon/gene ratio will be 1 and the normalized value (log_2_) will be 0. Then, the *splicing index* indicates that there is no splicing associated with the respective exon.

As shown in Figure 3A, a linear regression analysis allows defining a general trajectory for the expression of a specific gene-exon pair in a sample dataset. However, sometimes not all the samples included in a dataset fit well on a linear regression with respect to the majority of the samples. In our tissue samples study, when we plot the expression signal profile of a gene across the 33 samples (red line with dots in Figure 3B) and the profiles of all its exons (black and grey lines in Figure 3B), we observe that some samples show a deviation revealed by the separation between the profile corresponding to the gene and the profile corresponding to a specific exon (see red line *versus* black line in Figure 3B). As indicated in the previous section, for each sample we can calculate the deviation in expression against the estimated expression values calculated for each specific gene-exon pair by applying a linear regression model to the data. In this way we obtain the ***residual regression score*** calculated for each sample and each gene-exon pair (Figure 3C). This parameter is more accurate than the *splicing index* calculated to estimate specific changes in the exons of some samples with respect to the overall expression of the corresponding gene-exon pair. In fact, if we calculate the *splicing index* for one exon (ENSE00001527616) of the exemplary gene *RGN* (ENSG00000130988) using the dataset of 11 human tissues, we observe that the *splicing index* detects significant changes in at least 12 samples, since the normalized ratio between the exon expression signal and the gene expression signal (logNI) is > |0.5| for these 12 samples (Figure 3D). However, in the same example (i.e. exon ENSE00001527616 and gene ENSG00000130988) the results obtained calculating the ***residual regression score*** show that only 2 tissues, cerebellum (*cer*) and liver (*liv*) (i.e. 6 samples), have a clear change with respect to the expected signal of the exon (Figure 3C). This result is in line with an alternative splicing event previously reported for this gene between cerebellum and liver [8] and eliminates other putative alternative splicing events identified by the *splicing index* that would give false positive results. Although other methods previously published (and tested in this work) have been proved to be more robust than the *splicing index*, this example is illustrative of how the “probe” effects or other artificial sources of variability can result in false positive findings. Therefore, despite the need for a broader validation versus other methods, we can say that our method based on linear regression model is to be more precise and stringent than the *splicing index* approach and, when applied to the detection of changes in large-scale analyses of all the human exons (i.e. hundreds of thousands of exons), it will provide a smaller number of false positive results.

### Use of a reference set of human tissue-specific splicing genes

It has been estimated that approximately 95% of the human multi-exon genes may present several active isoforms, making a total expectation of approximately 100,000 splicing events in the human transcriptome [20]. However, there are not many genome-wide datasets that have experimentally proven the presence of alternative splicing and exon shuffling in human samples. A recent analysis of 15 diverse human tissue and cell line transcriptomes derived from deep-sequencing of cDNAs yielded an inventory of gene and mRNA isoform expression [8]. This study reports a large set of distinct human genes that show alternative splicing when comparing their expression in two different tissues. As described in Methods a large part of this dataset is referred in Figure 1, presenting the number of genes that show splicing in 15 pairwise comparisons of six human tissues/organs: breast, cerebellum, heart, liver, muscle and testes. The numbers indicate that some tissues include more genes with alternative isoforms than others (e.g. cerebellum). The total number of positive cases where these genes show splicing between two given tissues is 282. We used these genes to test the statistical sensitivity and specificity of the method here proposed (ESLiM) implemented in 3 slightly different versions (ESLiMa, ESLiMc, ESLiMt) described above. We also used the described reference set of human tissue-specific splicing events for comparative analyses of the performance of our method with 3 other previously published methods.

### Comparison of several methods for alternative splicing detection

Together with the reference set of 270 human genes that undergo alternative splicing in six different tissues, we used –as described above– an additional experimental exon microarray dataset (Human Exon 1.0 ST) applied to the same human tissues. This allows to test different methods and to compare the accuracy in the detection of known splicing events [21]. First, we tried our method in ESLiMa, ESLiMc and ESLiMt. In parallel, we also tried three published methods: FIRMA [15], ARH [16] and COSIE [17]. The most widely used is FIRMA (referred in 75 publications according to Google-Scholar in April 2014), which is based on an extension of the additive model of the RMA method [22].

**Figure 4:**
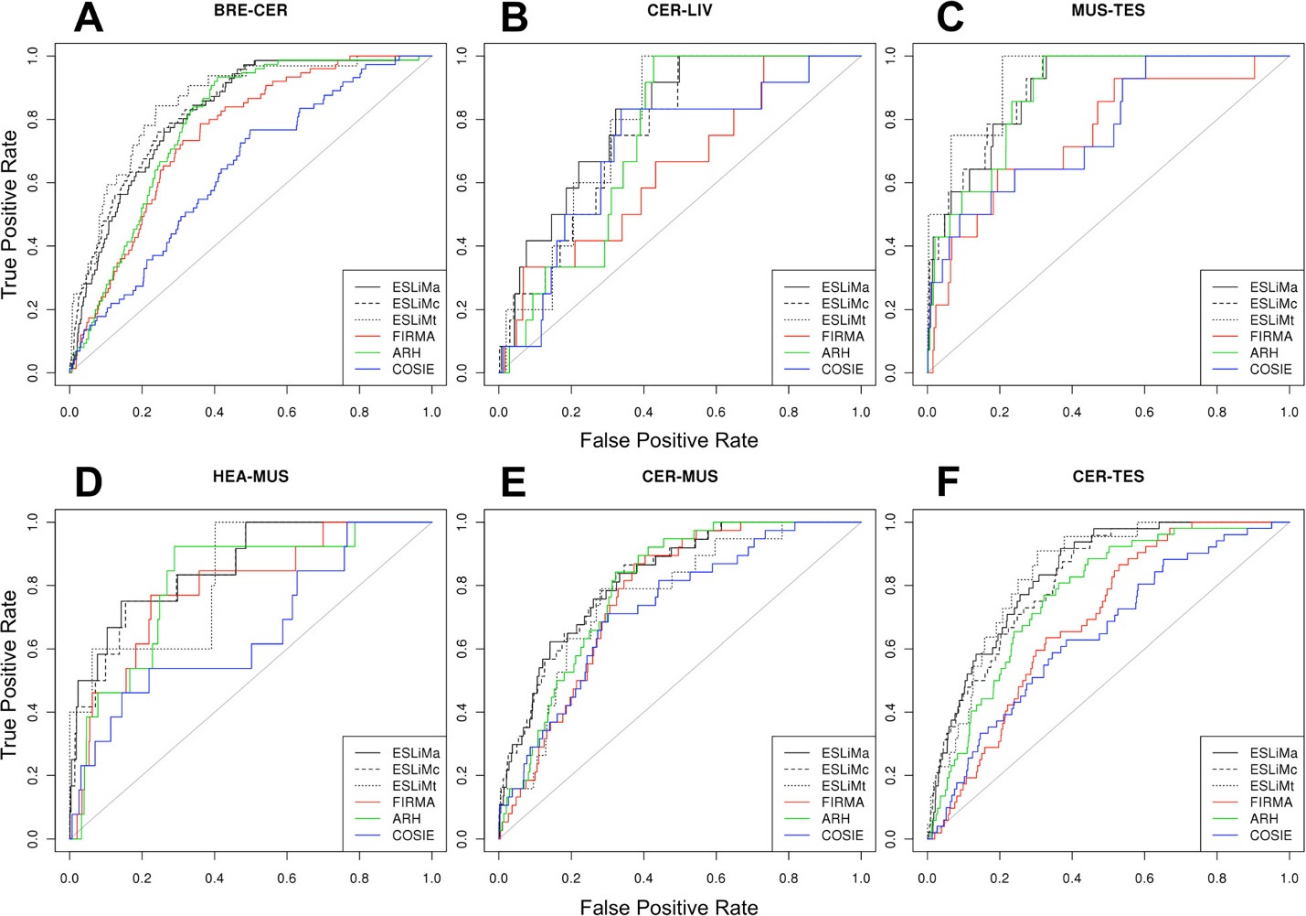
**ROC curves comparing the performance of five methods to identify splicing events in six tissue pairs.** The curves display the true positive rates versus the false positive rates (i.e. “sensitivity” versus “1–specificity”). The panels A-F correspond to the following tissue pairs: **(A)** BRE-CER breast and cerebellum; **(B)** CER-LIV cerebellum and liver; **(C)** MUS-TES muscle and testis; **(D)** HEA-MUS heart and muscle; **(E)** CER-MUS cerebellum and muscle; **(F)** CER-TES cerebellum and testis. A label indicating the color line that corresponds to each method is included in a box inside each plot: ESLiMa (black line), ESLiMc (black dashed line), ESLiMt (black dotted line), FIRMA (red), ARH (green) and COSIE (blue).

**Figure 5:**
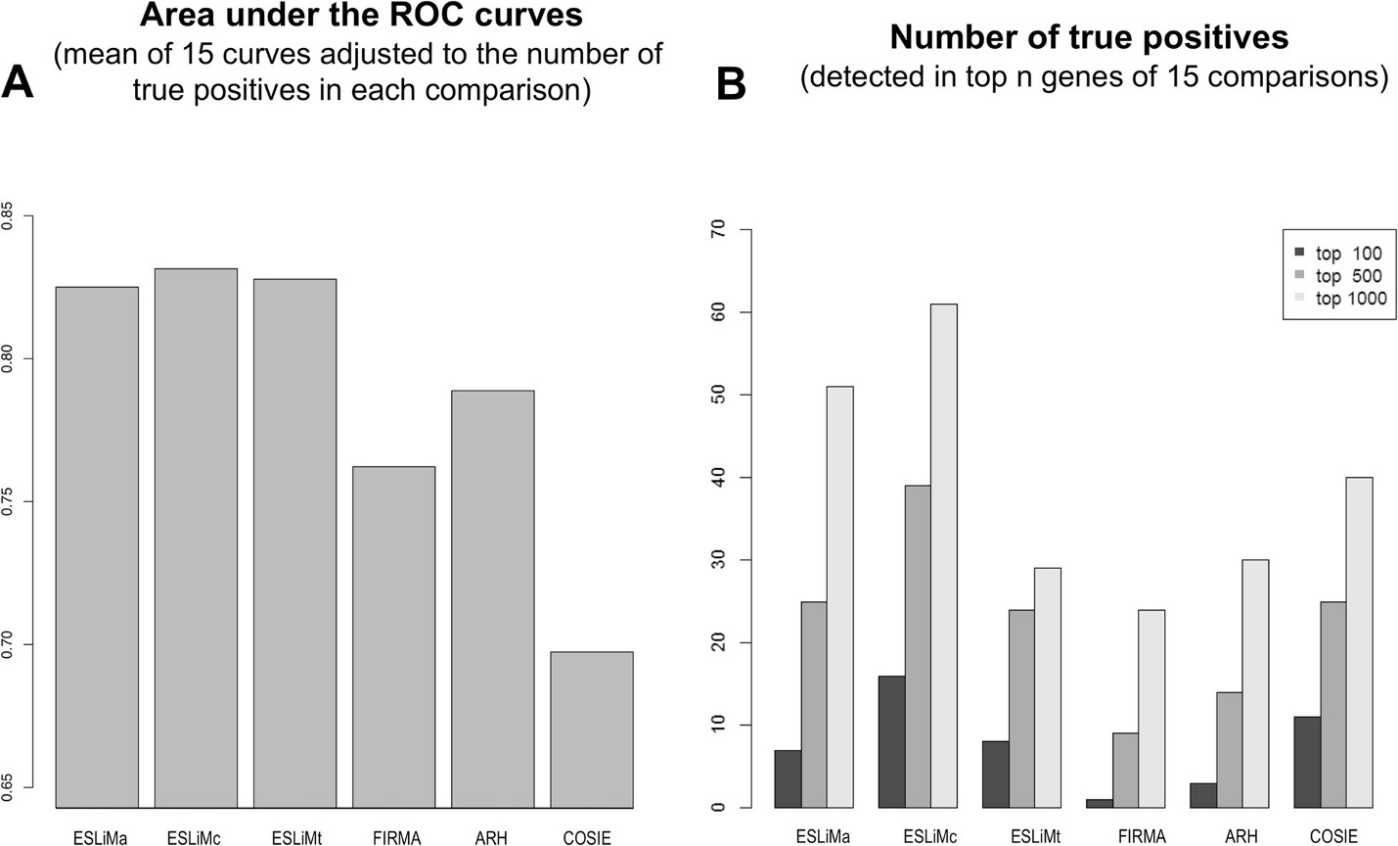
**AUC and number of true positives found by each method.** Barplots presenting the comparison of the performance of five methods to identify splicing events in 15 tissue pairs. **(A)** Area under the ROC curve (AUC) corresponding to each method considering the mean of 15 curves and adjusted to the number of true positives found in each comparison. **(B)** Number of true positives corresponding to each method detected in the top 100, 500, 1000 genes of the 15 tissue comparisons. The specific AUC values obtained for each of 15 comparisons corresponding to each one of the methods compared are provided in Additional file 2: Table S2.

The statistical analysis of the performance of all these methods is presented in Figure 4, which includes the ROC curves for six tissue pairs, and in Figure 5, which compares AUC values (area-under-the-curve) and number of true positives. In each case the number of true positives (TP) and true negatives (TN) were calculated using the reference set of human genes included in Figure 1. The ROC curves display the true positive rates (TPR = TP/(TP–FN)) *versus* the false positive rates (FPR = 1 – FP/(FP + TN)); that is equivalent to “sensitivity” *versus* “1–specificity”. ESLiMc (black dashed lines) is the method that performs better than all others providing an average value for AUC of 0.832 considering 15 tissue pair comparisons (Figure 5A). The AUC values obtained for these 15 comparisons corresponding to each one of the methods are provided in Additional file 2: Table S2. AUC averages were weighted by the number of validated splicing events in each comparison. The numbers of genes showing evidence for splicing are indicated in Figure 5B for each one of the tissue pair comparisons performed with each method (see also Additional file 3: Table S3). These genes are found in the top 100, 500 and 1000 most significant genes (i.e. top genes ranked by best p-values) detected by each method. Again ESLiMc is the method that shows the best performance.

In the ROC values we observed that the ESLiM approaches, compared to the other algorithms, present higher true positive rates (TPR) at low false positive rates (FPR). This feature can be very valuable when experimental validations are planned in accordance with the ranking of genes returned. In particular, ESLiMc is the best method in most cases. The lower performance of ESLiMt with respect to ESLiMc can be due to the fact that approximately only the 75% of the protein-coding genes can be measured with the restriction of finding probes in all known transcripts, and this reduces quite a lot the number of validated genes that can be detectable. The list of 61 spliced genes identified by ESLiMc in the top 1000 genes is provided as supplementary material (Additional file 4: Table S4), and they can be considered a revalidated set of human genes that present alternative splicing in different tissues, as identified in this work.

In the overall comparison with the other methodological approaches (i.e. ARH, COSIE, FIRMA) we can say that ESLiM improves findings in about 5-10% of the ROC values due to the application of the linear regression model. About another 2-5% of the ROC values improvement can be attributed to the application of the gene core signal calculated as described for ESLiMc. Comparisons considering other parameters, like the one in Figure 5B, also reflect a clear improvement provided by the application of the linear regression model. In fact, COSIE, the external method that performed best after ESLiMc for the detection of true positives (in the top 100 and top 500 spliced genes), is a method that includes a non-linear correlation model to calculate a corrected splicing index [17]. With regard to the other methods, we have already described the principles of gene-exon linear regression model compared to a splicing index (SI) approach; and most methods include small modifications of the SI calculation (like PAC, MIDAS, etc) [23]. In general, the comparisons presented in Figure 5 show a considerable improvement using ESLiMc; even with respect to FIRMA that is probably the most widely used method [15]. There are many additional tools and bioinformatic applications that have been published to help in the analyses of exon arrays datasets, like easyExon [24] Exon-Array-Analyzer [25], AltAnalyze [26] and BEAT [27]; but most of them are not very innovative approaches because they usually are integrated software packages including standard statistical methods (such us ANOVA and SI) or including other known algorithms (such us FIRMA [15] and MADS [28]). One final comment about these tools is that several of them allow the usage of different probesets-to-gene mappings (core, extended or full) provided by *Affymetrix* for the exon arrays; and most methods recommend to use the “core” mapping for splicing detection. However, it is important to realize that these exon array “core probesets” do not correspond with what we called the “gene core” included in ESLiMc, that uses only the probes that map on the exons conserved in long transcripts (covering ≥60% of the loci). The intention to improve on splicing detection may be similar, but the concept of “exons conservation” along transcripts and the practical way to calculate the “gene core” is specific to the method presented here.

Finally, it is worth to note that “sample size” is important for applying the ESLiM method given that it takes advantage of using all samples to do the linear regression estimates and to correct artificial probe effects and control the variability of genes showing different expression levels in different sample types. Therefore, in order to work properly, the method should be used with at least 20 or 30 different sample measurements. A dataset with very few replicates and only two different biological types will produce no significant result for exon changes in highly differentially expressed genes. The limitation of a “minimum sample size” (≈20-30 samples) does not mean that ESLiM is not adequate to do binary comparisons with just two biological classes. In these cases the method works fine as far as there are 10 to 15 samples for each class compared. This is shown with a practical example in the next section, proving that ESLiM produces coherent results in the comparison of two disease subtypes.

### Experimental validation of ESLiMc with an independent set of leukemia samples

In order to further evaluate the power of the method ESLiMc to identify specific exon re-arrangements and alternative splicing events, we analyzed a dataset of acute myeloid leukemia (AML) samples (n = 64) and mononuclear cell control samples (n = 6). As indicated above, the AML samples included two specific subtypes: **(i)** 24 core binding factor AMLs and **(ii)** 40 AMLs with complex karyotype. Using ESLiMc we did the analysis of splicing between these AML subtypes comparing the 40 CK-AMLs *versus* the 24 CBF-AMLs and identified, using a cutoff of FDR < 0.01, 1180 exons showing significant alteration (i.e. showing differential expression with adjusted p-values <10^-7^ and relative R-fold changes >1.5 for overexpression and <0.65 for repression) (data included in Additional file 6: Table S5). These exons correspond to 654 protein-coding genes, showing clear signs of a disease subtype-specific alternative splicing. In parallel, we checked the expression of transcripts in peripheral blood mononuclear cells (PBMNC) of healthy controls, to assess whether the detected transcripts were specific to AML. Based on these analyses, we confirmed splicing events by conventional RT-PCR in a selected number of 10 genes (Additional file 7: Table S6) out of 12 tested genes, observing a validation rate that shows a good performance of the algorithm on cancer samples. This subset of genes was selected for experimental validation due to a potential leukemogenic impact on the respective AML subtypes. Several of them have been correlated with AML pathology (like the leukemia-associated factor *RUNX3*/*AML2*), and are known to have multiple transcript variants resulting from alternative splicing events (like *ARHGAP4*, *EPHB6*, *MPG*, *PLXNB1*, *RUNX3* and *SEC14L1*; as indicated in NCBI GENE database).

**Figure 6:**
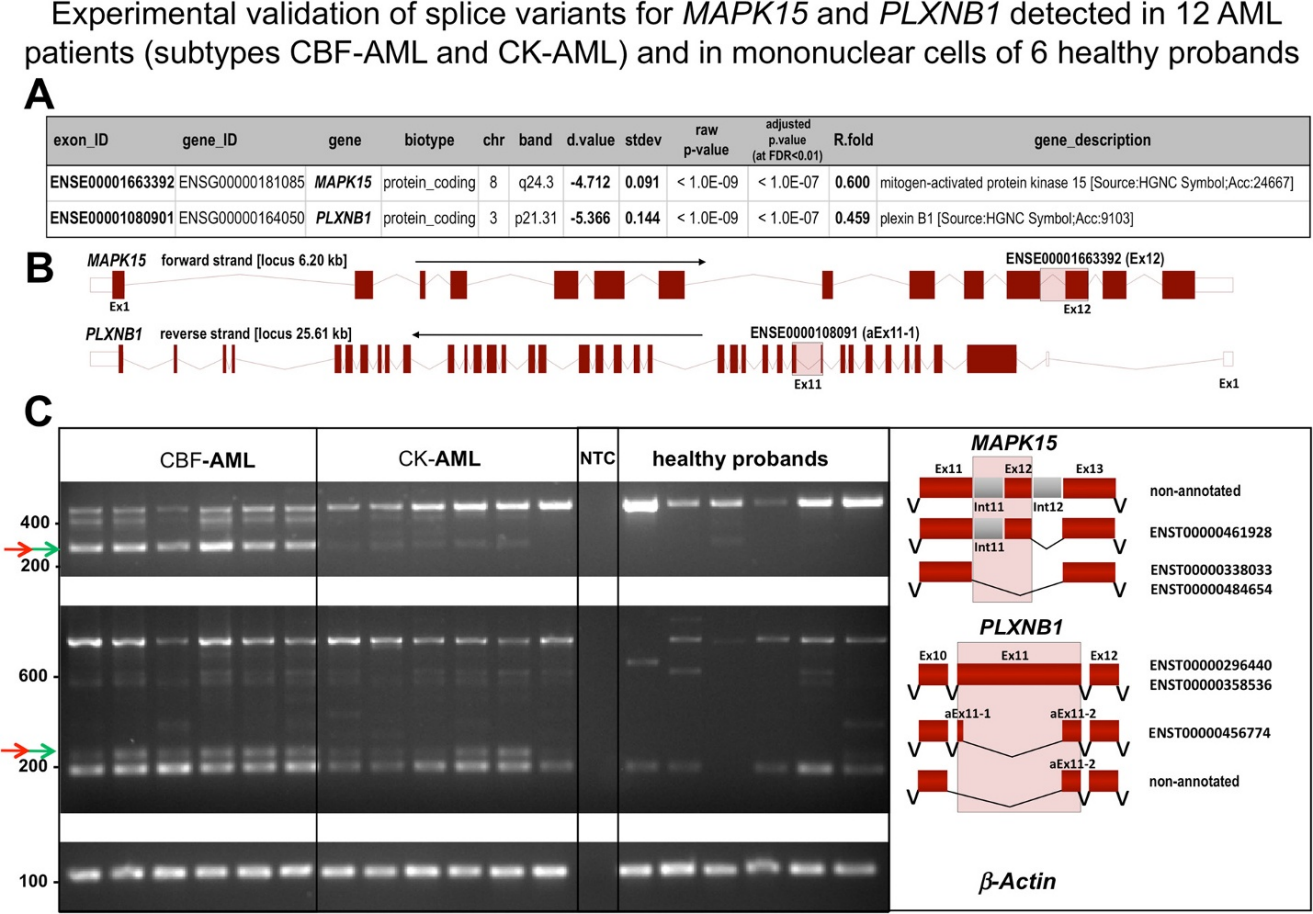
**Experimental validation of splicing patterns discriminating two subtypes of acute myeloid leukemia.** Validation of splice variants for genes *MAPK15* and *PLXNB1* detected in AML patients and in mononuclear cells of healthy controls. **(A)** Table with the output results provided by ESLiMc method showing the exons most significantly changed in genes *MAPK15* and *PLXNB1*. **(B)** Scaled pictures presenting the architecture of *MAPK15* and *PLXNB1* gene loci including all exons and introns. Red boxes show the coding exons; white boxes the untranslated regions (UTR); pink shadow boxes mark the exons which undergo splicing (ENSE00001663392 for *MAPK15* and ENSE0000108091 for *PLXNB1*). **(C)** Validation by RT-PCR performed with RNA samples from 12 AML patients (6 CBF-AML and 6 CK-AML) and with RNA from mononuclear cells of 6 healthy probands. The splice isoforms differentially expressed between the two AML subgroups are marked with red arrows. The splice isoforms differentially expressed between AML subgroups and the mononuclear blood fraction from healthy donors are marked with green arrows. NTC is for “no template control”. *Beta-actin* was used as loading control. The architecture of the specific exon/intron region from genes *MAPK15* and *PLXNB1* that show changes is presented aside.

For two of the most significant genes (*MAPK15* and *PLXNB1*) that showed alternative splicing, and that were most relevant for our AML study, we conducted a specific RT-PCR analysis to see if the reported alterations could be assigned to the indicated exons. For this validation 6 samples per subgroup were analyzed. In Figure 6 we present the experimental results for the exons detected as most changed for these two genes (ENSE00001663392 for *MAPK15* and ENSE0000108091 for *PLXNB1*, Figure 6A). For *MAPK15* gene (also called *ERK8*), which regulates localization of nuclear receptors and is involved in cellular proliferation and transformation [29], we could confirm the presence of a known isoform retaining a 137 bp intron in CBF samples that is not present in CK-AML or healthy control samples (isoform ENST00000461928/*MAPK15.fAug10* with EST accession number BX451569, previously found in human fetal brain cDNA library). Similarly, for plexin B1 (*PLXNB1*) gene, which encodes the semaphorin 4D receptor and has been shown involved in tumor progression of several cancers [30], we also could confirm the detection of previously annotated alternative transcripts (ENST00000296440 and ENST00000456774/*PLXNB1.dAug10*), as well as a novel alternative transcript (Figure 6C). This alternative isoform, detected as a 233 bp PCR product, was expressed in CBF-AML and CK-AML (though less in CK), and it was not expressed at all in healthy control cells (Figure 6C). Sanger sequencing of the PCR product revealed the loss of the exon (exon fragment aEx11-1) observed in the *PLXNB1* gene (data not shown). These two examples show that our novel algorithm provides a helpful tool to detect alternative splicing events using exon expression data and that the identified exon alterations can drive the way to discover relevant isoforms associated to the diseases studied.

**Figure 7:**
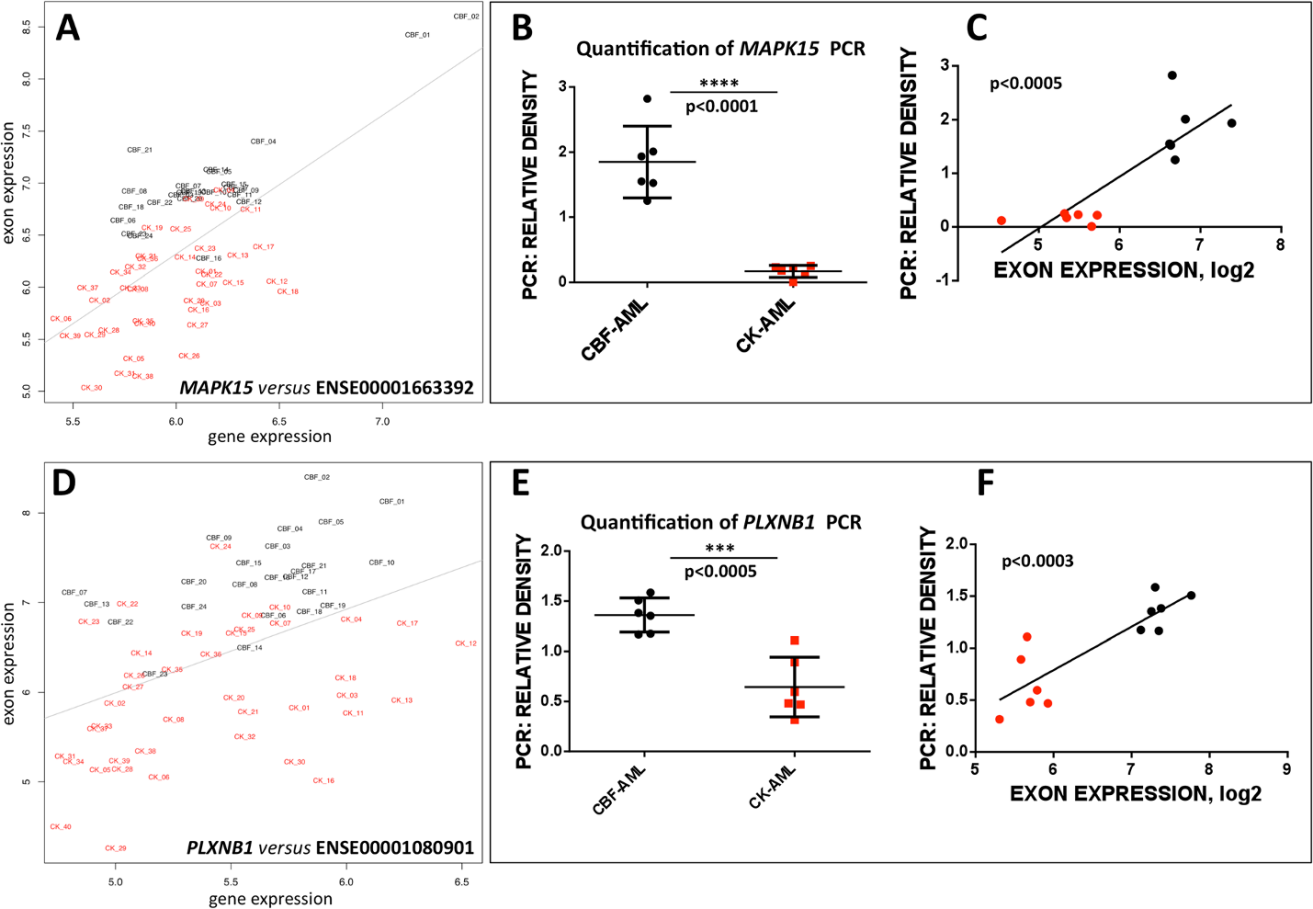
**Quantification of the relative expression of** ***MAPK15*** **and** ***PLXNB1*** **splice variants in two subtypes of acute myeloid leukemia.** **(A)** ESLiMc correlation plot of exon expression (exon ID: ENSE00001663392*)* of *MAPK15 versus* gene expression using the normalized expression signals calculated as indicated in Methods. **(B)** Quantification of *MAPK15* splice variant expression by measuring the relative density of *MAPK15* PCR product in ImageJ software (showing a significant differentially expression between CBF- and CK-AML). **(C)** Correlation plot of relative density of PCR product with ENSE00001663392 expression values. Relative density of PCR product is plotted along the *x*-axis and mean log_2_ transformed probeset intensities for ENSE00001663392 (*Affymetrix* probeset IDs 3119813, 3119814) are shown along *y*-axis. CK-AML (red dots) correspond to AML with complex karyotype, and CBF-AML (black dots) to core binding factor AML. **(D)** ESLiMc correlation plot of exon expression (exon ID: ENSE00001080901*)* of *PLXNB1 versus* gene expression using the normalized expression signals calculated as indicated in Methods. **(E)** Quantification of *PLXNB1* splice variant expression by measuring of PCR bands intensities in ImageJ software (this shows a significantly higher expression of *PLXNB1* splice isoforms in CBF-AML, which is in agreement with ESLiM correlation plot on D). **(F)** Correlation of relative PCR band intensities of *PLXNB1* splice variants with ENSE00001080901 exon expression. Relative density of PCR product is plotted along the *x*-axis and mean log_2_ transformed probe set intensities for ENSE00001080901 (*Affymetrix* probeset ID 2673238) are shown along *y*-axis.

Finally, it is worth to note that the experimental validation done with the AML samples is more qualitative than quantitative (i.e. as a proof of concept and singular testing of the method) since we do not pretend to include a full statistical analysis of this dataset in this work. Moreover, the specific genes validated (*MAPK15* and *PLXNB1*) were selected in the context of our current studies on the AML biology and not just randomly between all the significant results provided by ESLiMc. Nevertheless, quantification of findings showed a highly significant correlation with our ESLiM estimates (Figure 7), and thus the results are a proof of the value of the method in a real case, showing the detection of splicing events on two disease subtypes.

## Conclusions

In conclusion, our data suggest that the newly developed ESLiMc method (ESLiM-core) presented here provides a robust tool for the detection of alternative splicing events via the identification of specific exons which show significant relative changes –gain or loss– with respect to the genes. The algorithm outperforms several methods currently used, providing considerably better accuracy and it is suitable for validation of known splicing events as well as for exploration and discovery of novel non-annotated transcripts in human samples. The method is provided as an R package and can be applied to different types of samples and easily adapted to different types of datasets. Finally, the work shows that a correct definition of the gene core allows a better comparison of gene and exon expression signals and our strategy can help to improve the analyses of complex transcriptomic datasets [1, 7] that are still not well studied.

At last, we consider that the use of a “gold standard” dataset of genes in this work has been essential to achieve a good comparison and evaluation of several splicing detecting methods. The fact that this information was derived from RNA-seq was circumstantial, since our main concern was to have an independent and experimentally validated set of human genes undergoing splicing in different tissues. With respect to this point, it is important to indicate that most of the bioinformatic analytical methods are very much dependent on the specific technological platform that is used to measure the biological signals. It is clear that both expression arrays and RNA-seq measure genome-wide expression signals, but the way they do it –in technological detail– is totally different [31]. Microarrays measure the expression signal by hybridization on oligo “probes” that map each exon and each gene locus, but RNA-seq platforms measure the expression signal by sequencing and counting “reads” that map on each exon and each gene locus. Moreover, the quantification of the “probe-signals” and the “read-signals” at deep level needs the application of different algorithms. Despite this, we consider that some general principles and characteristics of the ESLiMc method can be transferred and applied to methods designed for the analysis of RNA-seq data. These applicable principles are: **(i)** the proposed approach to estimate whole gene signal that takes into consideration the exons conserved in the transcripts that cover a major part of the gene locus; **(ii)** the use of a linear regression modeling strategy to estimate the expression signal that is attributed to each exon/gene pair and to calculate the differential expression changes between them.

### Availability of supporting data

As indicated in Methods section the algorithm presented in this work (ESLiM) is provided as an R package together with examples of use and the mapping CDF files, all included as supporting data at URL (http://bioinfow.dep.usal.es/xgate/splicing/splicing.php): (Supporting data 1) ESLiMc R package: *ESLiM_1.0.tar.gz*; (Supporting data 2) Example of use for ESLiMc R package: *ESLIM_Install_and_Use.R*; (Supporting data 3) ExonMapper CDF R package: *exonmapperhumanexon1.0cdf_3.0.tar*; (Supporting data 4) ESLiMc GeneMapper CDF R package: package: *eslimcgenemapperhumanexon1.0cdf_3.0.tar.gz*. Moreover, we also provide at web site http://bioinfow.dep.usal.es/xgate/splicing/splicing.php the annotation R package for human exons (R data file: *exons.human.Annotation.RData*); and the dataset corresponding to the 33 exon microarrays GeneChip Human Exon 1.0 ST (file: *affy_dataset.zip*) containing 3 replicas of 11 different healthy human tissues.

## Electronic supplementary material

Additional file 1: Table S1: List of human genes reported by Wang *et al*. [8] that are alternatively spliced and were found in the pairwise tissue contrasts reported in Figure 1. The list includes 270 distinct genes identified in 282 pairwise contrasts. (XLS 72 KB)

Additional file 2: Table S2: AUC values obtained for 15 pairwise tissue comparisons and for each one of the six compared methods: ESLiMa, ESLiMc, ESLiMt, FIRMA, ARH and COSIE. AUC averages were weighted by the number of validated splicing events in each comparison. (XLS 17 KB)

Additional file 3: Table S3: Number of reference genes showing evidence for splicing for each one of the tissue pair comparisons performed with each method. These reference genes are found in the top-100, top-500 and top-1000 most significant genes detected by each method (ranked by best p-values). (XLS 10 KB)

Additional file 4: Table S4: List of 61 spliced genes identified by method ESLiMc as detected in the top-1000 genes provided by this method. The table includes the ENSEMBL ID, the gene symbol, the chromosomal location, the gene description and the tissue-pair where each gene has been detected. (XLS 28 KB)

Additional file 5: Table S7: Analysis of the architecture of known human protein-coding genes presenting the number of protein-coding transcripts (pcTranscripts) that cover different % of their locus length (≥T %). The data show that most (85%) of the transcripts that cover ≥60% of the human loci length correspond to stable and well-annotated protein-coding transcripts. The table also presents the number of gene loci covered by at least one transcript of a given locus length (i.e. ≥ that a given % of the locus length). The numbers indicate, for example, that 99.7% of the human gene loci included at least one protein-coding transcript that covered ≥60% of its locus length. (XLS 14 KB)

Additional file 6: Table S5: Analysis of splicing detected in two AML subtypes, done using ESLiMc method: 40 samples of CK-AMLs *versus* 24 samples of CBF-AMLs. For a cutoff of FDR<0.01 1180 exons were found showing significant alteration. These exons were found considering: differential expression with adjusted p-values <10^-7^ and relative R-fold changes >1.5 for overexpression and <0.65 for repression. These exons correspond to 654 distinct protein-coding genes. (XLS 350 KB)

Additional file 7: Table S6: Splicing events that were confirmed by conventional RT-PCR in a selected number of 10 human genes. The table presents the values provided by the analysis done using ESLiMc method. For these 10 genes, a total of 22 exons were detected as statistically significant. (XLS 44 KB)
